# Ni_0.85_Se Nanosheet Arrays on Nickel Foam as a Robust Non-Noble Metal Electrocatalyst for Energy-Efficient Urea Oxidation

**DOI:** 10.3390/nano16211357

**Published:** 2026-10-25

**Authors:** John Anthuvan Rajesh, Shrine Maria Nithya Jeghan, Soon-Hyung Kang, Kwang-Soon Ahn

**Affiliations:** 1 School of Chemical Engineering, Yeungnam University, Gyeongsan 712-749, Republic of Korea; 21500915@ynu.ac.kr (J.A.R.); shrine@ynu.ac.kr (S.M.N.J.); 2 Department of Chemistry Education, Chonnam National University, Gwangju 500-757, Republic of Korea; skang@jnu.ac.kr

**Keywords:** Ni_0.85_Se, nanosheets, hydrothermal, electrocatalyst, urea oxidation

## Abstract

We report the facile synthesis of Ni_0.85_Se nanosheet arrays directly grown on nickel foam (NF) via a two-step hydrothermal strategy using Ni(OH)_2_ as a precursor. The two-dimensional architecture offers abundant electroactive sites and efficient charge transport pathways, resulting in remarkable electrocatalytic activity toward the urea oxidation reaction (UOR). The optimized Ni_0.85_Se/NF electrode delivers a current density of 10 mA cm^−2^ at a potential of 1.352 V vs. RHE and exhibits a small Tafel slope of 42.3 mV dec^−1^. Furthermore, it demonstrates excellent durability, maintaining stable operation for over 120 h in alkaline urea-containing electrolyte. These findings highlight Ni_0.85_Se nanosheets as a cost-effective and durable non-noble-metal electrocatalyst with significant potential for urea oxidation application.

## 1. Introduction

The oxygen evolution reaction (OER) and urea oxidation reaction (UOR) are two pivotal anodic processes in electrochemical energy conversion and environmental remediation technologies [1,2]. The OER involves a sluggish four-electron transfer pathway and requires a high overpotential, which limits the overall efficiency of water electrolysis for hydrogen production. In contrast, the UOR occurs at a much lower potential, thereby significantly reducing the energy input required for hydrogen generation while simultaneously enabling the degradation of urea-containing wastewater [1,2,3,4,5]. Hence, the development of efficient, low-cost, and durable electrocatalysts for both OER and UOR is of great importance for advancing sustainable hydrogen-based energy systems [6,7].

Noble-metal catalysts such as IrO_2_, RuO_2_, and Pt exhibit excellent catalytic activity toward OER and UOR. However, their high cost and scarcity impede large-scale applications, prompting extensive research into earth-abundant alternatives [1,2,3,4,5]. Transition metal–based materials, particularly those containing Ni, Co, and Fe, have been widely explored in the form of oxides, hydroxides, phosphides, sulfides, and selenides as potential candidates for UOR electrocatalysis [8,9,10,11,12,13]. Among them, Ni-based catalysts have attracted considerable attention due to their ability to generate high-valence Ni^3+^/Ni^4+^ species that serve as active centers for urea oxidation. Moreover, nickel-based materials are inexpensive, earth-abundant, and exhibit favorable redox properties in alkaline media [14,15,16,17].

Recently, nickel selenides (e.g., NiSe, Ni_0.85_Se, NiSe_2_, and Ni_3_Se_2_) have emerged as promising bifunctional electrocatalysts for both OER and UOR due to their enhanced electronic conductivity, adjustable electronic structures, and strong metal–selenium interactions [18,19,20,21,22,23,24,25]. Selenium incorporation modulates the local electronic configuration of nickel, facilitating charge transfer and improving the adsorption/desorption dynamics of key reaction intermediates. In particular, Ni_0.85_Se offers an optimized Ni/Se ratio that enhances electrical conductivity and exposes abundant Ni^2+^/Ni^3+^ redox sites, both of which are critical for superior electrocatalytic activity [24,25,26].

Furthermore, tailoring Ni_0.85_Se into a nanosheet-like morphology provides additional advantages, such as an enlarged electrochemically active surface area, enhanced mass/electron transport, and improved structural robustness [26,27,28]. Nanosheet arrays on conductive nickel foam (NF) ensure intimate contact between the active phase and current collector, leading to reduced interfacial resistance and rapid charge transfer during catalysis [26].

In this study, we present a simple two-step hydrothermal synthesis of Ni_0.85_Se nanosheet arrays directly grown on NF, with Ni(OH)_2_ serving as a precursor. The resulting Ni_0.85_Se/NF electrode exhibits an interconnected nanosheet architecture and excellent structural integrity. Benefiting from its optimized composition and morphology, the electrode requires a potential of only 1.352 V vs. RHE to achieve a current density of 10 mA cm^−2^, exhibits a small Tafel slope of 42.3 mV dec^−1^, and maintains long-term stability for 120 h at 100 mA cm^−2^ in alkaline urea solution. These results demonstrate that Ni_0.85_Se/NF is a highly efficient and durable non-noble-metal electrocatalyst with significant potential for efficient urea oxidation application.

## 2. Experimental

### 2.1. Materials

Nickel nitrate hexahydrate (Ni(NO_3_)_2_.6H_2_O), urea (CH_4_N_2_O), ammonium fluoride (NH_4_F), sodium selenite (Na_2_SeO_3_), hydrazine hydrate (N_2_H_4_·H_2_O), and potassium hydroxide (KOH) were obtained from Sigma-Aldrich and used without further purification. Ethanol (C_2_H_6_O) and deionized (DI) water were used as solvents throughout the experiments. NF (1.6 mm thick) was purchased from MTI Corporation, Seoul, Republic of Korea, and served as both the conductive substrate and the nickel source for the catalyst growth.

### 2.2. Synthesis of Ni_0.85_Se Nanosheets

Ni_0.85_Se nanosheets were synthesized via a simple two-step hydrothermal process. In the first step, nickel hydroxide (Ni(OH)_2_) nanosheets were directly grown on NF to serve as a precursor. A precursor solution containing 50 mM Ni(NO_3_)_2_.6H_2_O, 250 mM urea, and 125 mM NH_4_F was prepared in 20 mL of DI water. A pre-cleaned piece of NF (1 × 5 cm^2^) was immersed in the solution, transferred to a 50 mL Teflon-lined stainless-steel autoclave, and maintained at 120 °C for 6 h. After cooling naturally to room temperature, the obtained Ni(OH)_2_/NF sample was thoroughly rinsed several times with DI water and ethanol to remove residual ions and then dried in an oven at 70 °C for 12 h.

In the second step, the Ni(OH)_2_ precursor was converted into Ni_0.85_Se through a controlled selenization process. Briefly, 0.1 g of Na_2_SeO_3_ was dissolved in 20 mL of DI water, followed by the slow addition of 1 mL of hydrazine hydrate under continuous stirring for 30 min to form a homogeneous solution. The prepared solution, together with the Ni(OH)_2_/NF sample, was transferred into a 100 mL Teflon-lined autoclave and heated at 120 °C for 12 h. After completion, the resulting Ni_0.85_Se/NF electrode was collected, thoroughly washed with DI water and ethanol several times, and dried at 70 °C overnight.

Details of materials characterization and electrochemical measurements are provided in the Supplementary Materials.

## 3. Results and Discussion

**Figure 1:**
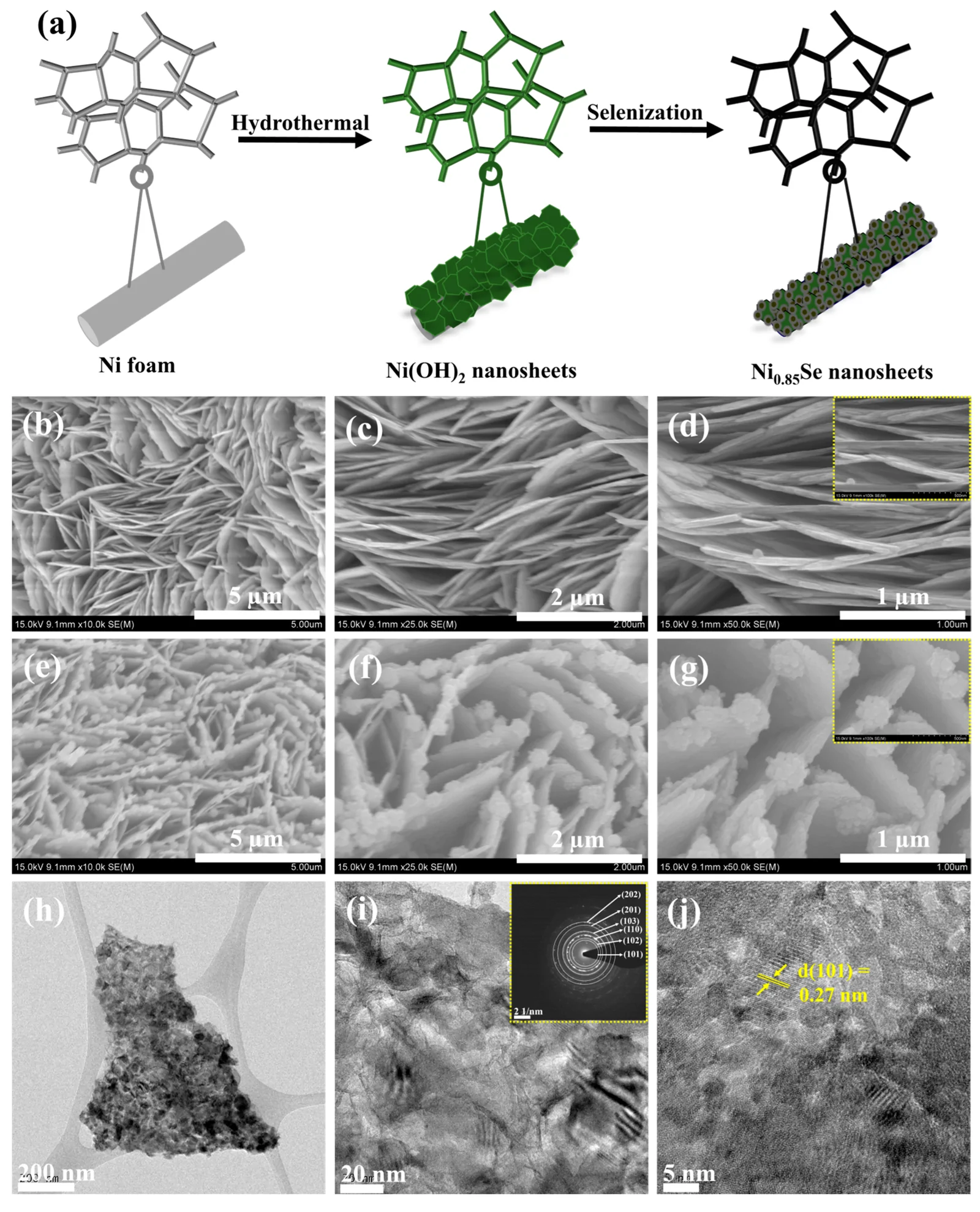
(**a**) Schematic illustration of the synthesis process of Ni_0.85_Se nanosheets grown on NF through a hydrothermal selenization route. (**b**–**d**) FESEM images of Ni(OH)_2_ nanosheets. (**e**–**g**) FESEM images of Ni_0.85_Se nanosheets. (**h**–**j**) TEM and HRTEM images of Ni_0.85_Se nanosheets.

The Ni_0.85_Se nanosheet structures were synthesized via a carefully designed two-step hydrothermal process, as illustrated in Figure 1a. In the first step, a uniform layer of Ni(OH)_2_ nanosheets was directly grown on the NF substrate. The formation of the green Ni(OH)_2_ precipitate occurs through the hydrolysis of the Ni(NO_3_)_2_.6H_2_O precursor, as represented by the following reaction:

Ni^2+^ + 2OH^−^ → Ni(OH)_2_ ↓ (1)

This process yields a dense and well-interconnected network of nanosheets, ensuring intimate contact between the active material and the conductive substrate.

In the second step, the as-prepared Ni(OH)_2_ precursor was converted into Ni_0.85_Se via a selenization treatment in the presence of hydrazine hydrate, which acts as both a reducing and structure-directing agent. The reactions involved can be described as follows:

2SeO_3_^2−^ + 3N_2_H_4_ → 2Se^2−^ + 3N_2_ + 6H_2_O (2)

0.85Ni^2+^ + Se^2−^ → Ni_0.85_Se (3)

During this conversion, the overall nanosheet morphology was well preserved, and the structure remained strongly anchored to the underlying NF, as shown in Figure 1a. The resulting architecture offers a high electrochemically active surface area and facilitates efficient electron and ion transport, which are highly beneficial for subsequent electrocatalytic reactions.

The morphology and structural features of the Ni(OH)_2_ precursor and Ni_0.85_Se electrocatalyst were analyzed using field-emission scanning electron microscopy (FESEM) and field-emission transmission electron microscopy (FETEM). As shown in Figure 1b–d, the Ni(OH)_2_ precursor exhibits a typical sheet-like morphology consisting of interconnected and loosely stacked dense nanosheets with lateral dimensions of several micrometers. The wrinkled surfaces and open network structure provide high surface area, abundant edge sites, and good mechanical stability—favorable characteristics for subsequent selenization and electrochemical reactions. Elemental mapping of the precursor (Figure S1a–c) reveals a uniform distribution of Ni and O, while the corresponding EDX spectrum (Figure S1d) confirms the absence of impurities, indicating high chemical purity of the Ni(OH)_2_ nanosheets. After the selenization process, the morphology largely retains its sheet-like structure, as shown in Figure 1e–g, though the nanosheets become slightly thicker and rougher. The increased surface roughness and compactness are attributed to the topotactic anion exchange between OH^−^ and Se^2−^ ions. This structural modification enhances surface activity and provides improved electron and ion transport channels, which are beneficial for catalytic processes. FESEM-based elemental mapping of the selenized product (Figure S2a–c) shows homogeneous distribution of Ni and Se, and the EDX spectrum (Figure S2d) confirms the presence of these two elements without oxygen-related impurities. These results verify the successful conversion of Ni(OH)_2_ into Ni_0.85_Se while maintaining structural integrity and uniform elemental dispersion. The strong adhesion of the nanosheets to the NF substrate ensures excellent electrical conductivity and mechanical stability during electrocatalysis.

The FETEM images provide deeper insight into the microstructural characteristics of the Ni_0.85_Se nanosheets. The low-magnification TEM image (Figure 1h) clearly displays the thin and wrinkled sheet-like morphology, indicating that the nanosheets are flexible and well-interconnected, consistent with the FESEM observations. This morphology facilitates efficient electrolyte penetration and provides abundant active sites for electrochemical reactions. The high-resolution TEM image (Figure 1j) reveals distinct lattice fringes with an interplanar spacing of 0.27 nm, which corresponds to the (101) plane of the hexagonal Ni_0.85_Se phase [26,28]. The well-defined fringes reflect the high crystallinity and uniform lattice ordering within the nanosheets. Furthermore, the selected-area electron diffraction (SAED) pattern (Figure 1i) shows multiple sharp and concentric diffraction rings indexed to the (101), (102), (110), (103), (201), and (202) planes, confirming the polycrystalline nature of Ni_0.85_Se and exhibiting excellent agreement with the XRD data [26,28]. Overall, the FESEM and FETEM analyses confirm that Ni_0.85_Se nanosheets were successfully formed through complete selenization of Ni(OH)_2_, yielding a highly crystalline, rough-surfaced architecture ideal for enhanced electrocatalytic performance in the urea-assisted oxygen evolution reaction.

**Figure 2:**
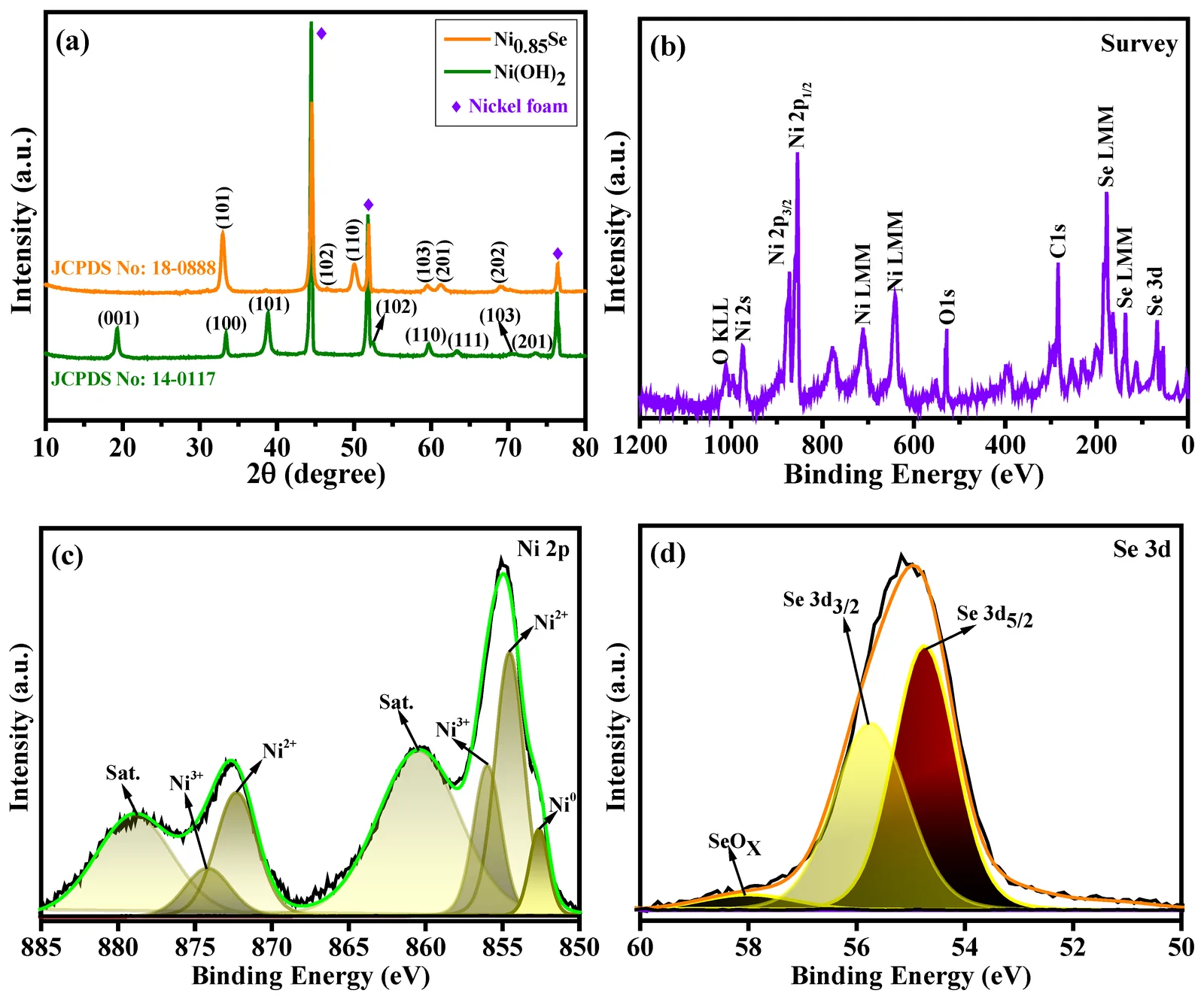
(**a**) XRD patterns of Ni_0.85_Se/NF and Ni(OH)_2_ electrocatalysts. (**b**) XPS survey spectrum of Ni_0.85_Se. (**c**) High-resolution Ni 2p spectrum. (**d**) High-resolution Se 3d spectrum.

The crystalline structure of both precursor and final product was analyzed by X-ray diffraction (XRD). As shown in Figure 2a, the diffraction pattern of the Ni(OH)_2_ precursor exhibits peaks that can be indexed to the brucite-type hexagonal phase of Ni(OH)_2_ (JCPDS No. 14-0117), confirming the successful formation of the hydroxide phase [29]. After the selenization step, the diffraction peaks corresponding to Ni(OH)_2_ disappear and a new set of reflections emerges at 2θ = 33.0° (101), 46.5° (102), 50.1° (110), 59.5° (103), 61.2° (201), and 69.7° (202). These peaks are in excellent agreement with those of the hexagonal Ni_0.85_Se phase (JCPDS No. 18-0888) [26], confirming the complete conversion of Ni(OH)_2_ into Ni_0.85_Se. The sharp and well-defined diffraction peaks indicate high crystallinity, while the absence of impurity or secondary phases demonstrates the phase purity of the synthesized material. This structural transformation highlights that the hydrothermal–selenization approach is highly effective for producing crystalline nickel selenide with controlled morphology.

The surface electronic states of the Ni_0.85_Se nanosheets were further examined by X-ray photoelectron spectroscopy (XPS). The survey spectrum (Figure 2b) confirms the presence of Ni and Se as the major elements, along with minor O and C signals. The O 1s peak can be attributed to surface oxidation of the selenide or adsorbed oxygen-containing species, while C arises mainly from adventitious hydrocarbons on the surface. The high-resolution Ni 2p spectrum (Figure 2c) displays two main peaks located at 854.9 eV (Ni 2p_3/2_) and 872.6 eV (Ni 2p_1/2_), each accompanied by characteristic satellite peaks. Upon deconvolution, the peaks at 854.7 eV and 872.4 eV are assigned to Ni^2+^ species, whereas those at 856.3 eV and 874.2 eV correspond to Ni^3+^ species [26,27,30]. In addition, a minor component observed at lower binding energy at 852.6 eV indicates the presence of metallic Ni^0^. The coexistence of Ni^0^, Ni^2+^, and Ni^3+^ confirms the mixed-valence nature of the material, which is known to promote enhanced redox activity and more efficient charge-transfer behavior during electrocatalytic processes. The Se 3d spectrum (Figure 2d) displays a spin–orbit doublet at 54.1 eV (Se 3d_5/2_) and 55.7 eV (Se 3d_3/2_), characteristic of Se^2−^ anions bonded to Ni in the Ni–Se lattice [26,27,30]. In addition, a weak feature around 58.1 eV corresponds to oxidized Se species (SeO_x_), which typically form on the surface upon exposure to air [30].

Overall, the XRD and XPS analyses collectively verify the complete transformation of Ni(OH)_2_ into phase-pure, crystalline Ni_0.85_Se nanosheets. The well-retained nanosheet morphology, high crystallinity, and coexistence of mixed Ni^2+^/Ni^3+^ oxidation states are expected to provide abundant active sites and improved electrical conductivity. Such structural and electronic features are crucial for promoting efficient charge transport and catalytic activity, making the synthesized Ni_0.85_Se nanosheets highly promising for electrocatalytic applications such as the urea oxidation reaction.

**Figure 3:**
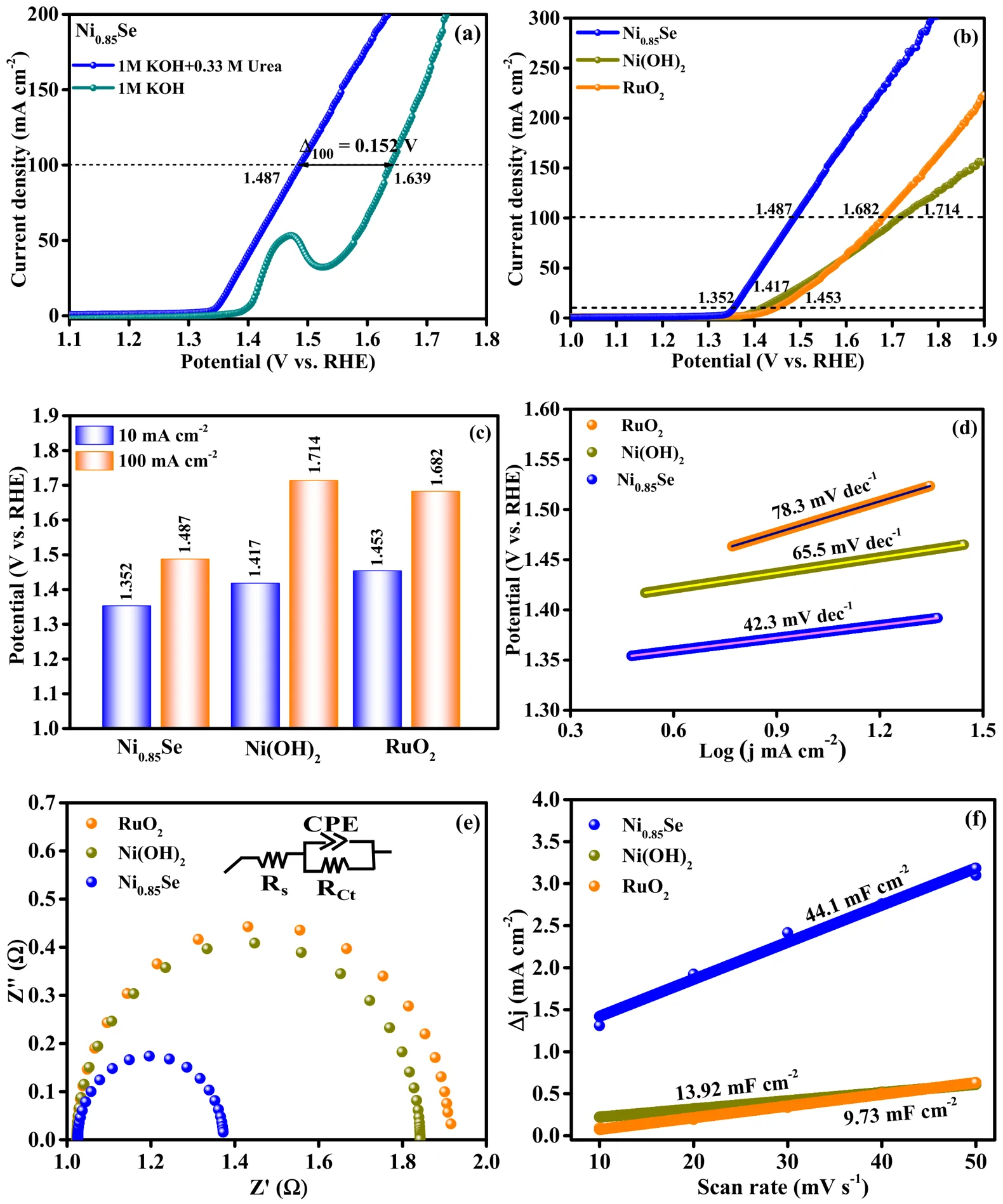
(**a**) LSV curves of Ni_0.85_Se/NF in 1 M KOH and 1 M KOH + 0.33 M urea. (**b**) Comparison of UOR polarization curves for Ni_0.85_Se, Ni(OH)_2_, and RuO_2_ electrocatalysts. (**c**) Comparison of the potentials required to achieve current densities of 10 and 100 mA cm^−2^ for different catalysts. (**d**) Tafel plots. (**e**) Nyquist plots obtained from EIS. (**f**) Double-layer capacitance (Cdl) extracted from CV curves.

The electrochemical performance of Ni_0.85_Se, Ni(OH)_2_, and RuO_2_ was evaluated in 1.0 M KOH + 0.33 M urea electrolyte using a standard three-electrode configuration, where a carbon rod, Hg/HgO, and NF-supported catalyst served as the counter, reference, and working electrodes, respectively. Prior to testing, each electrode was activated by cyclic voltammetry (CV) for 15 cycles at a scan rate of 50 mV s^−1^ to achieve surface stabilization. Subsequently, linear sweep voltammetry (LSV) measurements were carried out at a slower scan rate of 2 mV s^−1^ over a potential window of 1.0–1.8 V vs. RHE to determine the steady-state current density. Figure 3a compares the LSV curves of Ni_0.85_Se in 1.0 M KOH and 1.0 M KOH + 0.33 M urea. After activation, the LSV profiles exhibit a marked difference between OER and UOR regions. The pronounced oxidation peak observed in KOH corresponds to the OER, whereas the presence of urea significantly shifts the onset potential to a lower value, indicating a substantial reduction in potential for the UOR process. To achieve a benchmark current density of 100 mA cm^−2^, Ni_0.85_Se required a potential of 1.487 V vs. RHE in the urea-containing electrolyte, which is lower than that required for OER (1.639 V vs. RHE). This comparison demonstrates the favorable electrochemical behavior of Ni_0.85_Se toward UOR in the presence of urea-containing alkaline electrolyte. The enhanced UOR activity arises from the preferential oxidation of urea molecules prior to catalyst self-oxidation, which promotes the in-situ formation of high-valence NiOOH species [2,3,4,5]. These NiOOH phases act as the true active centers, facilitating rapid charge transfer and accelerating the overall reaction kinetics.

To further understand the UOR performance, all catalysts were evaluated under identical conditions, as shown in Figure 3b. The LSV curves of Ni_0.85_Se, Ni(OH)_2_, and RuO_2_ in 1.0 M KOH + 0.33 M urea clearly reveal that Ni_0.85_Se exhibits the highest catalytic activity. The onset potentials for Ni_0.85_Se, Ni(OH)_2_, and RuO_2_ were 1.338, 1.369, and 1.413 V, respectively. The lower onset potential of Ni_0.85_Se is attributed to the reduced electronegativity of selenium compared to oxygen, which effectively tunes the electronic structure of nickel sites, enhancing intrinsic catalytic activity. These results highlight the beneficial role of Se incorporation in modulating the charge distribution and optimizing adsorption energies for urea and its intermediates. Meanwhile, Ni(OH)_2_ exhibited superior activity compared to commercial RuO_2_ due to its nanosheet morphology, which provides abundant exposed Ni active sites that facilitate faster electron transfer. The superior performance of Ni-based catalysts compared to RuO_2_ can also be ascribed to the facile Ni^2+^/Ni^3+^ redox transitions that occur spontaneously during catalysis.

To further compare the UOR performance, the potentials required to achieve current densities of 10 and 100 mA cm^−2^ were extracted from the corresponding LSV curves (Figure 3b). At 10 mA cm^−2^, Ni_0.85_Se/NF required a potential of 1.352 V vs. RHE, which is lower than those of Ni(OH)_2_/NF (1.417 V vs. RHE) and RuO_2_ (1.453 V vs. RHE). Similarly, at 100 mA cm^−2^, Ni_0.85_Se/NF required 1.487 V vs. RHE, whereas RuO_2_ and Ni(OH)_2_/NF required 1.682 and 1.714 V vs. RHE, respectively (Figure 3c). These results demonstrate that Ni_0_**_._**_85_Se/NF exhibits favorable UOR activity across the investigated current-density range. The enhanced performance can be attributed to the synergistic effects of Se-induced electronic modulation, accessible Ni active sites, and efficient charge-transfer characteristics. Furthermore, the UOR activity of Ni_0.85_Se/NF is comparable to that of several previously reported Ni-based electrocatalysts (Table 1). For example, Ni_0.85_Se/rGO nanocrystals required a potential of 1.36 V vs. RHE [30], Ni_-_Se nanoparticles exhibited 1.347 V vs. RHE [31], and NiSe_2_@MoSe_2_ required 1.36 V vs. RHE [32] at similar current densities. These results highlight the promising UOR activity and stability of Ni_0.85_Se/NF, suggesting its potential as a cost-effective catalyst for urea oxidation.

Reaction kinetics were further probed using Tafel slope analysis (Figure 3d). Ni_0.85_Se exhibited the smallest slope (42.3 mV dec^−1^), compared to Ni(OH)_2_ (65.5 mV dec^−1^) and RuO_2_ (78.3 mV dec^−1^). The markedly reduced slope confirms that Ni_0.85_Se accelerates charge transfer and facilitates more favorable reaction pathways, further demonstrating the synergistic role of Se in enhancing intrinsic kinetics. Electrochemical impedance spectroscopy (Figure 3e) supports this conclusion, as Ni_0.85_Se exhibited the lowest charge-transfer resistance (Rct) 0.347 Ω, in stark contrast to the sluggish interfacial conductivity of Ni(OH)_2_ (0.817 Ω) and RuO_2_ (0.888 Ω). The reduced semicircle radius in the Nyquist plot reflects superior electron transport during UOR, emphasizing the conductivity advantage imparted by Se incorporation. The fitted EIS parameters, including the solution resistance (R_s_), Rct, and constant phase element (CPE) parameters, together with the corresponding fitting errors for all samples, are summarized in Table S1. The electrochemical double-layer capacitance (Cdl) was used to estimate surface-related charge storage behavior (Figure 3f). CV curves were recorded at different scan rates (10–50 mV s^−1^) in the non-Faradaic region to assess the intrinsic activity of the catalysts (Figure S3). Ni_0.85_Se recorded a Cdl of 44.1 mF cm^−2^, far exceeding those of Ni(OH)_2_ (13.92 mF cm^−2^) and RuO_2_ (9.73 mF cm^−2^). The higher Cdl value indicates a greater electrochemically active interface, which provides more accessible catalytic sites for urea adsorption and subsequent oxidation.

**Figure 4:**
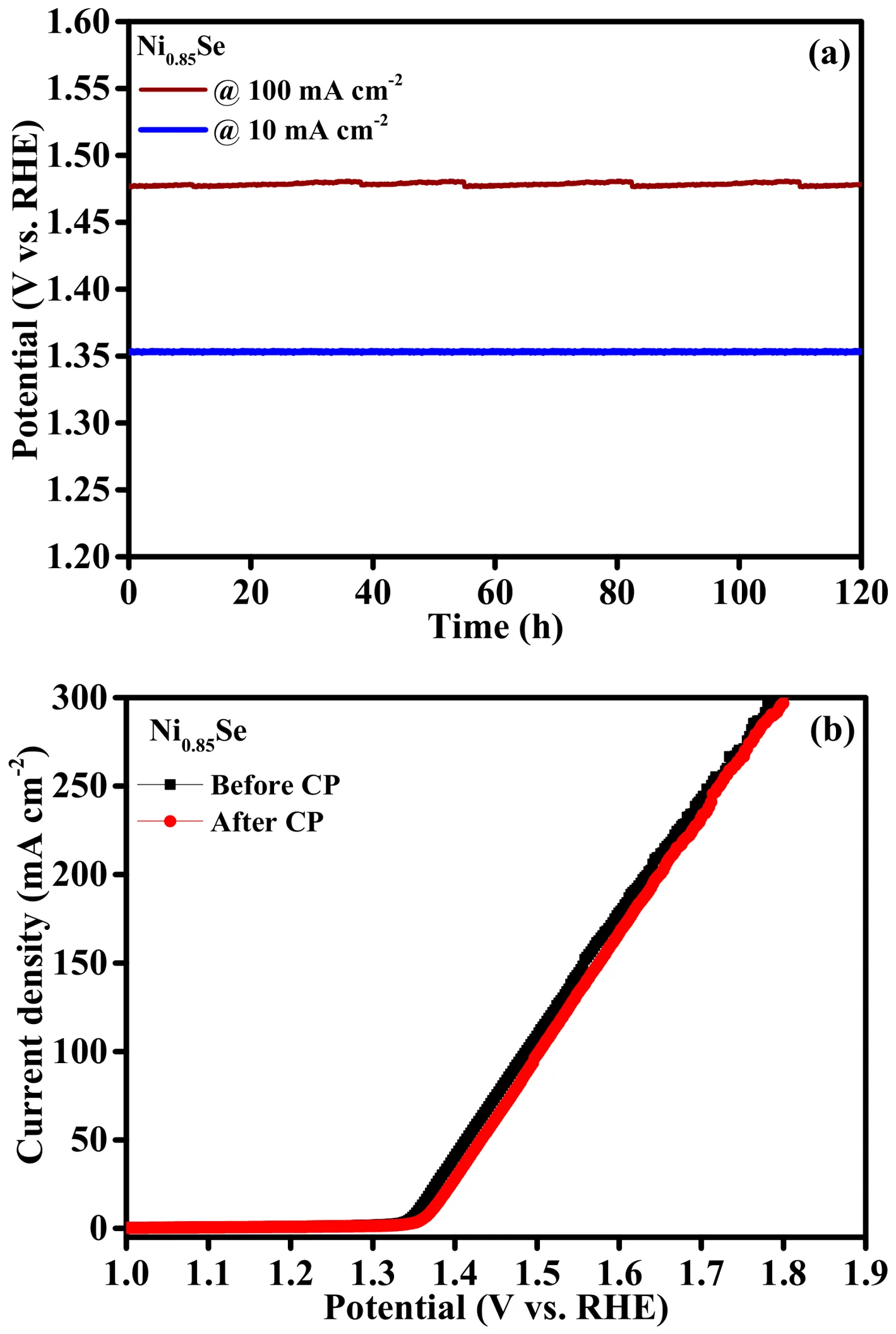
(**a**) Chronopotentiometry stability test of Ni_0.85_Se/NF at 10 and 100 mA cm^−2^ over 120 h. (**b**) LSV curves recorded before and after CP.

Durability was evaluated through chronoamperometry at 10 and 100 mA cm^−2^ for 120 h (Figure 4a). Ni_0.85_Se maintained nearly all of its catalytic activity with negligible current decay. Furthermore, the LSV curves before and after long-term testing showed only minor deviations (Figure 4b), confirming structural robustness. This outstanding stability arises from strong Ni–Se bonding, which stabilizes the catalyst framework, and the persistent formation of NiOOH, which mitigates dissolution and surface reconstruction under prolonged electrolysis [25].

**Figure 5:**
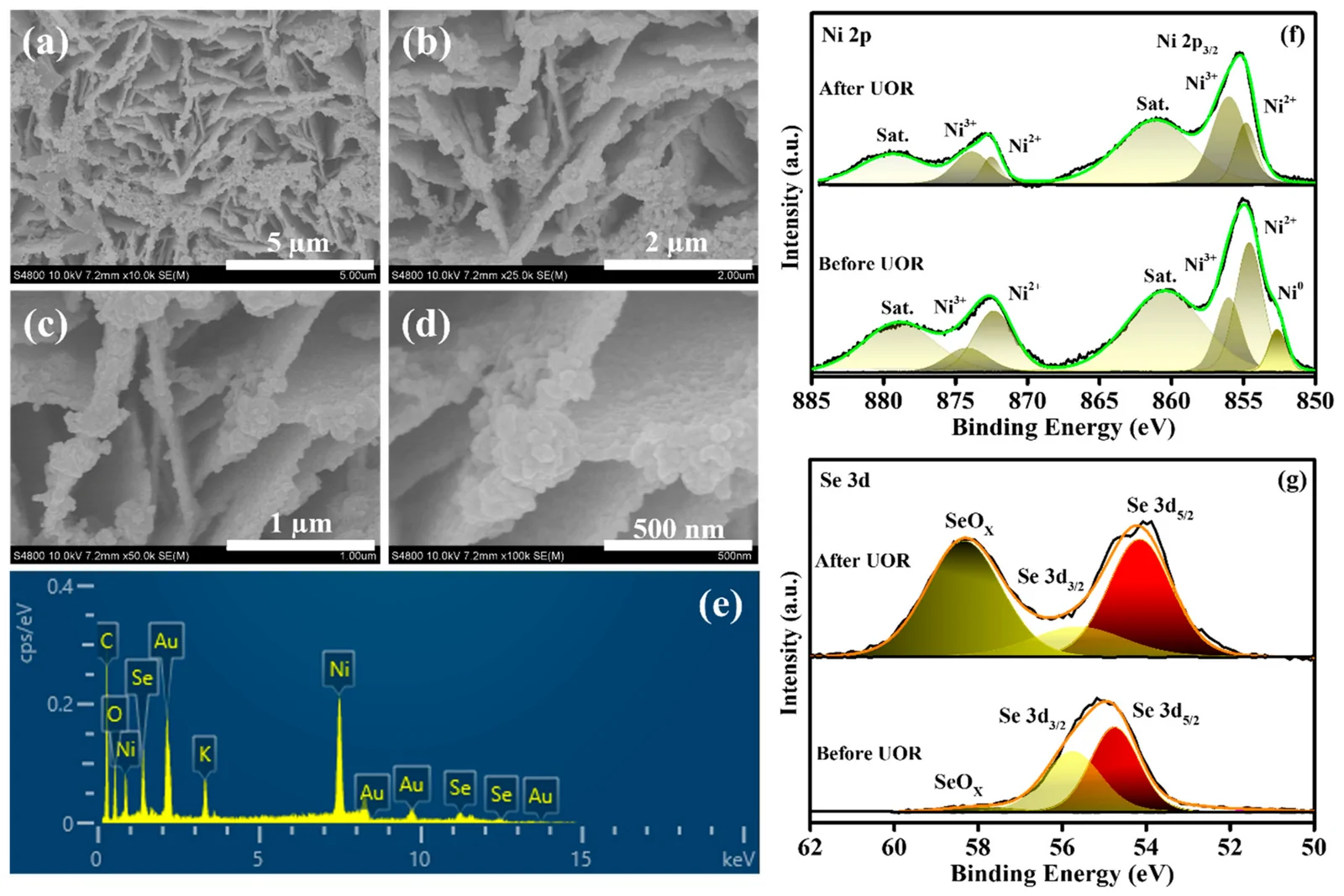
(**a**–**d**) FESEM images of Ni_0.85_Se nanosheets after the prolonged CP stability test. (**e**) Corresponding EDX spectrum. (**f**) Deconvoluted Ni 2p spectra before and after UOR. (**g**) Deconvoluted Se 3d spectra before and after UOR.

After chronoamperometric stability testing at 100 mA cm^−2^ for 120 h, the Ni_0.85_Se nanosheets largely retained their original sheet-like morphology, indicating excellent structural robustness (Figure 5a–d). Minor surface contamination was observed, which is commonly attributed to the adsorption of electrolyte species, carbonates, or trace impurities from the urea-containing alkaline medium. The EDX spectroscopy of the post-electrolysis electrode revealed a slight increase in oxygen and carbon content compared to the pristine sample (Figure 5e). The enhanced oxygen signal can be ascribed to partial surface oxidation of Ni_0.85_Se, forming NiOOH or other nickel oxy/hydroxide species during prolonged anodic polarization—consistent with the in-situ generation of active Ni^3+^ species that facilitate urea oxidation [24]. The slight increase in carbon content is likely due to adsorption of adventitious carbon from the electrolyte or environmental exposure during handling, which is frequently observed after extended electrochemical testing. Overall, these observations indicate that although minor surface changes occur, the bulk nanosheet structure remains intact, and the primary Ni–Se framework is preserved. Notably, the formation of surface NiOOH may even contribute positively to sustained catalytic activity by providing additional active sites for the UOR without significant structural degradation.

To further elucidate enhanced UOR activity, ex situ XPS analysis was performed on Ni_0.85_Se before and after the UOR process (Figure 5f,g). The Ni 2p spectra exhibits two main peaks at 855.2 eV and 872.6 ± 0.2 eV, corresponding to Ni 2p_3/2_ and Ni 2p_1/2_, respectively, along with their satellite peaks at 860.9 eV and 879.1 eV. The deconvoluted peaks at binding energies of 854.7 eV/872.3 eV and 856.0 eV/873.7 eV are attributed to Ni^2+^ and Ni^3+^ species, respectively. After the UOR process, the intensity of the Ni^3+^ peaks increased significantly compared to the Ni^2+^ signals, confirming oxidation of Ni^2+^ to Ni^3+^ and indicating active electron transfer during catalysis [24,25]. Similarly, the Se 3d spectra reveal the emergence of Se–O species after UOR, suggesting partial surface oxidation of selenium. The reduction in the Se 3d intensity, coupled with the enhancement of Ni 2p signals, confirms effective participation of both Ni and Se species in the redox process.

Mechanistically, the superior UOR performance of Ni_0.85_Se can be attributed to three synergistic factors: (i) in situ generation of high-valence NiOOH species that serve as active centers, (ii) Se-induced electronic modulation that lowers the energy barrier for urea adsorption and intermediate oxidation, and (iii) enhanced electrical conductivity and structural stability provided by the robust Ni–Se framework. Collectively, these findings demonstrate that Ni_0.85_Se exhibits outstanding activity, rapid kinetics, and long-term durability—outperforming conventional catalysts such as Ni(OH)_2_ and RuO_2_—thereby establishing it as a highly efficient and earth-abundant catalyst for practical urea oxidation applications.

## 4. Conclusions

In summary, Ni_0.85_Se derived from Ni(OH)_2_ demonstrates promising bifunctional activity toward both OER and UOR in alkaline media. The catalyst requires potentials of 1.352 and 1.487 V vs. RHE to achieve current densities of 10 and 100 mA cm^−2^, respectively, demonstrating its favorable UOR performance. Compared with Ni(OH)_2_ and RuO_2_, Ni_0.85_Se exhibits a smaller Tafel slope, reduced charge-transfer resistance, and higher Cdl, underscoring the beneficial role of Se incorporation. Moreover, excellent long-term stability is maintained over 120 h of continuous operation. These results demonstrate the potential of Ni_0.85_Se as a durable and earth-abundant non-noble-metal electrocatalyst for efficient urea oxidation and related environmental applications.

## Figures and Tables

**Table 1:** Comparison of electrochemical UOR performance of Ni_0.85_Se/NF with previously reported electrocatalysts.

Electrocatalysts	Morphology	Potential (V)	Tafel Slope (mV dec^−1^)	Electrolyte	Ref.
**Ni_0.85_Se/NF**	**Nanosheets**	**1.352 (10 mA** **cm^−2^** **)** **1.487 (100 mA** **cm^−2^** **)**	**42.3**	**1 M KOH + 0.33 M urea**	**This work**
NiSe-Ni_3_Se_2_/GNF	Nanoparticles	1.39 (50 mA cm^−2^) 1.41 (100 mA cm^−2^)	36.9	1 M KOH + 0.33 M urea	[20]
Ni_0.85_Se/rGO	Nanocrystals	1.36 (10 mA cm^−2^)	152.08	1 M KOH + 0.5 M urea	[30]
Ni_0.85_Se/NiTe	Core–shell structure	1.301 (100 mA cm^−2^)	3.66	1 M KOH + 0.33 M urea	[33]
Ni-Se	Nanoparticles	1.347 (10 mA cm^−2^)	102.1	1 M KOH + 0.33 M urea	[31]
Ni-TPA@NiSe	Nanosheets@ nanorods	1.37 (100 mA cm^−2^)	22.7	1 M KOH + 0.5 M urea	[34]
Ni_3_Se_4_/NiSe_2_	Nanowrinkles	1.296 (100 mA cm^−2^)	44.4	1 M KOH + 0.5 M urea	[35]
Cr_0.25_-NiSe_2_	Nanosheets	1.37 (100 mA cm^−2^)	13.2	1 M KOH + 0.33 M urea	[36]
NiSe@Ni_12_P_5_/NCF	Nanospheres@ nanorods	1.267 (10 mA cm^−2^) 1.412 (100 mA cm^−2^)	53.65	1 M KOH+ 0.5 M urea	[37]
NiSe_2_@MoSe_2_	Spherical clusters	1.36 (10 mA cm^−2^)	55.1	1 M KOH + 0.5 M urea	[32]

## Data Availability

The data used is already included in the manuscript.

## Supplementary Materials

The following supporting information can be downloaded at: https://www.mdpi.com/article/10.3390/nano16211357/s1, Experimental parts such as, materials characterization and electrochemical measurements. Figure S1. (a) FESEM image of Ni(OH)_2_ nanosheets. (b, c) Elemental mappings of Ni and O. (d) Corresponding EDX spectrum. Figure S2. (a) FESEM image of Ni_0.85_Se nanosheets. (b, c) Elemental mappings of Ni and Se. (d) Corresponding EDX spectrum. Figure S3. Typical CV curves recorded at different scan rates in the non-Faradaic region for (a) Ni_0.85_Se/NF, (b) Ni(OH)_2_/NF, and (c) RuO_2_ electrocatalysts. Table S1. EIS-fitted parameters for Ni_0.85_Se, Ni(OH)_2_ and RuO_2_ electrocatalysts.
