# Supplementary material for: Ni0.85Se Nanosheet Arrays on Nickel Foam as a Robust Non-Noble Metal Electrocatalyst for Energy-Efficient Urea Oxidation
Source: Nanomaterials (Basel). 2026 Oct 25;16(21):1357. doi: 10.3390/nano16211357 (PMC13764941; doi:10.3390/nano16211357)
Supplement: Supplementary file 1 [file nanomaterials-16-01357-s001.zip › nanomaterials-4597480-supplementary.pdf]

nanomaterials

Supplementary Information

Article

$Ni_{0.85}Se$ Nanosheet Arrays on Nickel Foam as a Robust Non-Noble Metal Electrocatalyst for Energy-Efficient Urea Oxidation

John Anthuvan Rajesh [1,†], Shrine Maria Nithya Jeghan [1,†], Soon-Hyung Kang [2] and Kwang-Soon Ahn [1,*]

[1] School of Chemical Engineering, Yeungnam University, Gyeongsan 712-749, Republic of Korea; 21500915@ynu.ac.kr (J.A.R.); shrine@ynu.ac.kr (S.M.N.J.)
[2] Department of Chemistry Education, Chonnam National University, Gwangju 500-757, Republic of Korea; skang@jnu.ac.kr
* Correspondence: kstheory@ynu.ac.kr; Tel.: +82-53-810-2524; Fax: +82-53-810-4631
† These authors contributed equally to this work.

Materials characterization

The crystalline phases of the samples were identified by X-ray diffraction (XRD) using a PANalytical X'Pert Pro diffractometer equipped with Cu Kα radiation (λ = 1.54060 Å), operated at 40 kV and 30 mA. Surface chemical states were analyzed by X-ray photoelectron spectroscopy (XPS, Thermo Scientific K-Alpha ESCA) with monochromated Al Kα radiation. Morphological features were examined using field-emission scanning electron microscopy (FE-SEM, Hitachi S4800) and field-emission transmission electron microscopy (FE-TEM, FEI Tecnai G2 F20). The elemental composition and distribution were further confirmed by energy-dispersive X-ray spectroscopy (EDS, Oxford Instruments).

Electrochemical Measurements

The electrocatalytic performance of the synthesized $Ni_{0.85}Se$ nanosheets toward the oxygen evolution reaction (OER) and urea oxidation reaction (UOR) was systematically evaluated using an IVIUMSTAT electrochemical workstation (Netherlands) in a standard three-electrode configuration. A graphite rod was employed as the counter electrode, an Hg/HgO electrode as the reference electrode, and $Ni_{0.85}Se$ coated on nickel foam ($Ni_{0.85}Se$/NF) served as the working electrode. For comparison, $Ni(OH)_2$/NF and commercial RuO_2 electrodes were also tested under identical conditions. All measurements were performed in 1.0 M KOH + 0.33 M urea electrolyte.

Linear sweep voltammetry (LSV) was performed at a scan rate of 2 mV s^{-1} within the potential range of 1.0 to 1.8 V vs. RHE. The corresponding polarization curves were used to evaluate catalytic activity, while the Tafel slope values were derived to analyze the reaction kinetics. The double-layer capacitance (C_{dl}) was estimated from cyclic voltammetry (CV) curves recorded at various scan rates (10–50 mV s^{-1}) in the non-faradaic region (0.72–0.78 V vs. RHE). The difference between the anodic and cathodic current densities, divided by two [$(j_{anodic} - j_{cathodic})/2$] at 0.78 V (vs. RHE), was plotted against the scan rate. The slope of the linear relationship was used to determine the corresponding C_{dl} value.

Electrochemical impedance spectroscopy (EIS) was conducted in the frequency range of 100 kHz to 1 Hz with an AC perturbation of 5 mV to evaluate charge-transfer resistance and interfacial kinetics. The long-term durability of $Ni_{0.85}Se$/NF was examined by

chronopotentiometry (CP) at a constant current density of 10 and 100 mA cm^{-2} for 120 hours in UOR condition.

All electrode potentials reported in this study are referenced to the reversible hydrogen electrode (RHE). For UOR performance evaluation, the experimentally measured potentials required to achieve specified current densities are directly reported without converting them into overpotentials.

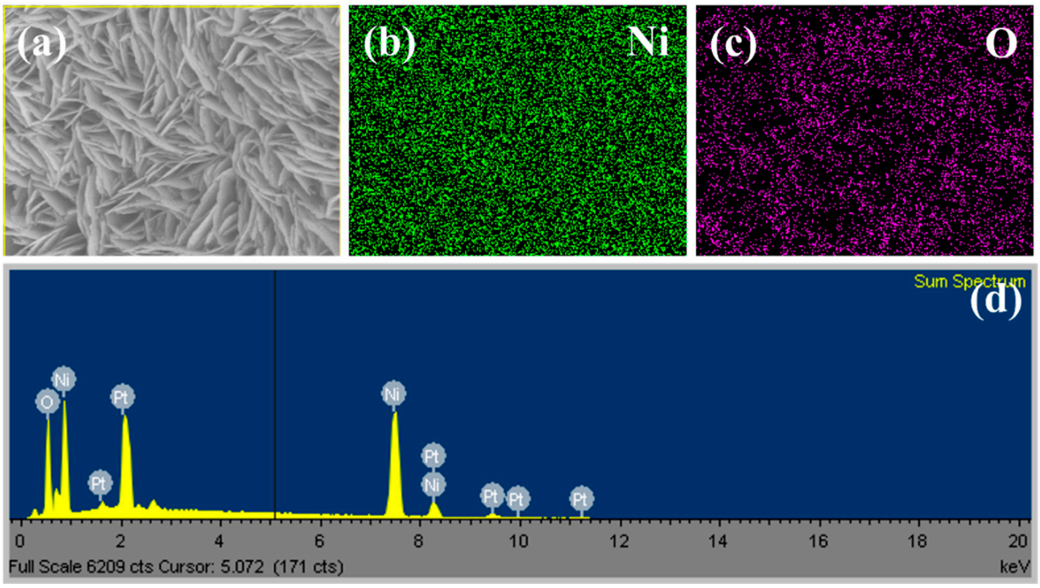

Figure S1. (a) FESEM image of $Ni(OH)_2$ nanosheets. (b, c) Elemental mappings of Ni and O. (d) Corresponding EDX spectrum.

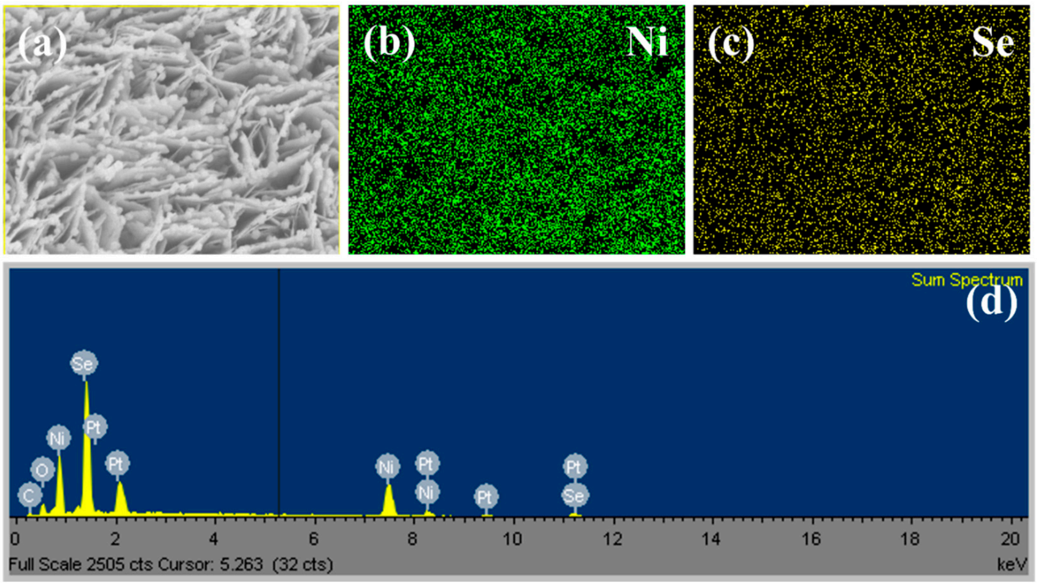

Figure S2. (a) FESEM image of $Ni_{0.85}Se$ nanosheets. (b, c) Elemental mappings of Ni and Se. (d) Corresponding EDX spectrum.

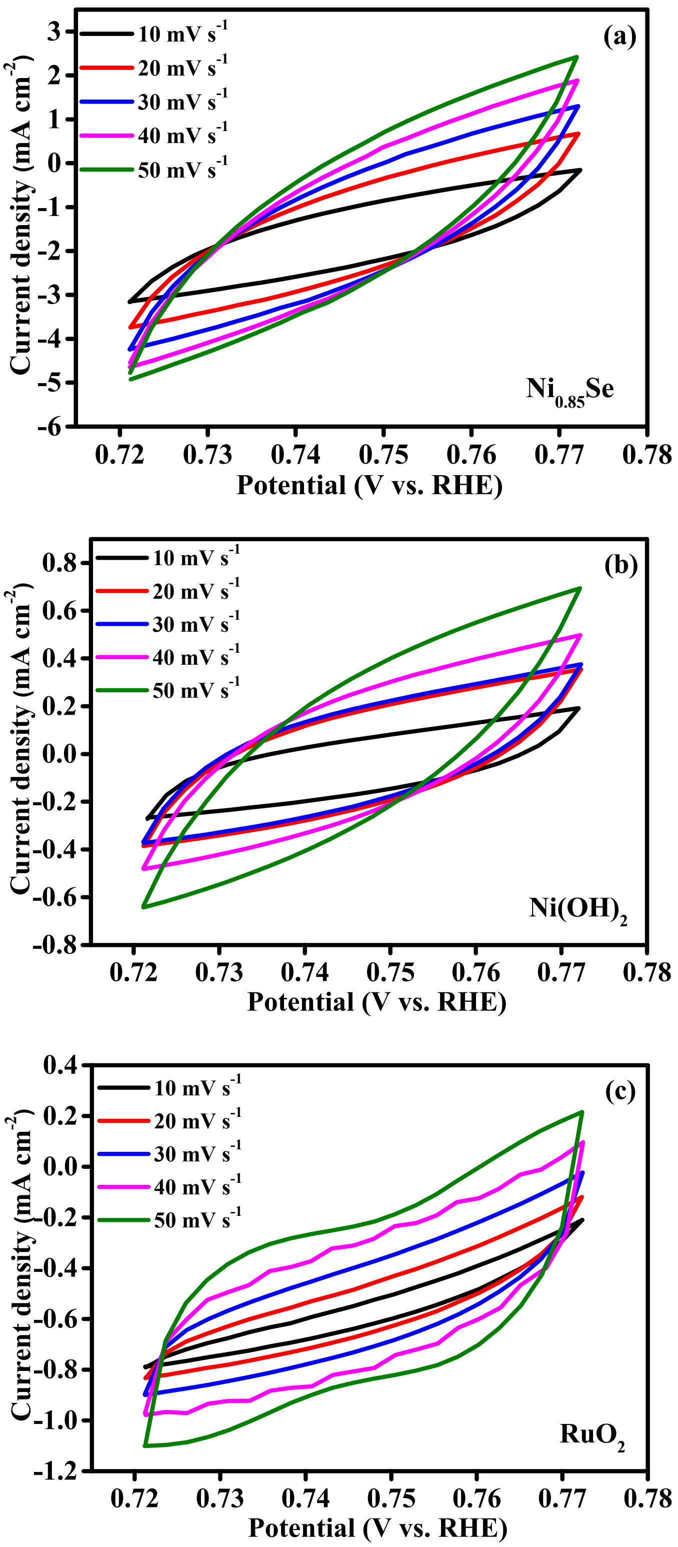

Figure S3. Typical CV curves recorded at different scan rates in the non-Faradaic region for (a) $Ni_{0.85}Se$/NF, (b) $Ni(OH)_2$/NF, and (c) RuO_2 electrocatalysts.

Table S1. EIS-fitted parameters for $Ni_{0.85}Se$, $Ni(OH)_2$ and RuO_2 electrocatalysts.

Electrocatalysts	R_s (Ω)	Right intercept (Ω)	R_{ct} (Ω)	CPE (F)
$Ni_{0.85}Se$	1.02551 ± 0.004	1.37224	0.34746 ± 0.0010	0.209803 ± 0.0021
$Ni(OH)_2$	1.02393 ± 0.006	1.84172	0.81779 ± 0.009	0.138015 ± 0.004
RuO_2	1.02342 ± 0.004	1.9117	0.88831 ± 0.0011	0.115975 ± 0.004
